# Radiofrequency Ablation vs. Surgical Resection for Small Early-Stage Primary Intrahepatic Cholangiocarcinoma

**DOI:** 10.3389/fonc.2020.540662

**Published:** 2020-09-29

**Authors:** Xin Xiang, Daixing Hu, Zheng Jin, Pan Liu, Huapeng Lin

**Affiliations:** ^1^Department of Hepatobiliary Surgery, The First People's Hospital of Neijiang, Neijiang, China; ^2^Department of Endocrine and Breast Surgery, The First Affiliated Hospital of Chongqing Medical University, Chongqing, China; ^3^Department of Gastroenterology, Affiliated Hangzhou First People's Hospital, Zhejiang University School of Medicine, Hangzhou, China; ^4^Department of Intensive Care Unit, Affiliated Hangzhou First People's Hospital, Zhejiang University School of Medicine, Hangzhou, China

**Keywords:** radiofrequency ablation, intrahepatic cholangiocarcinoma, early-stage, survival, SEER

## Abstract

**Aim:** We aimed to compare the survival outcomes of radiofrequency ablation (RFA) and surgical resection (SR) for patients with small early-stage primary intrahepatic cholangiocarcinoma (ICC).

**Methods:** Patients with small (≤5 cm) and early-stage ICC were screened from the Surveillance, Epidemiology, and End Results (SEER) database. Overall survival (OS) and cancer-specific survival (CSS) rates between the SR and RFA patients were evaluated. The results were verified using an inverse probability-weighting model (IPTW).

**Results:** In total, 184 patients with small T1 stage ICC that received RFA or SR treatment were identified. The OS rates at 1, 3, and 5 years were 87.4, 73.3, and 61.5% for patients who underwent SR, respectively, and 89.9, 42.4, and 23.9%, respectively, for patients who received RFA. CSS rates at 1, 3, and 5 years were 91.5, 73.8, and 66.1%, respectively, for the SR group and 93.5, 53.4, and 30.0%, respectively, for the RFA group. The OS and CSS rates were evaluated to be significantly better in the SR group than in the RFA group after the multivariate Cox regression and IPTW analysis. Subsequently, the survival benefit of SR was also observed in the subgroup of patients with <4.5 or <4 cm early-stage ICC when compared with RFA.

**Conclusion:** Our results indicated that the SR provided a significantly better prognosis than RFA in patients with small and early-stage ICC. SR as the first-line treatment of primary early-stage ICC is still recommended. However, prospective randomized controlled trials with larger sample sizes are required to compare these modalities in the treatment of ICC.

## Introduction

Intrahepatic cholangiocarcinoma (ICC), a subtype of cholangiocarcinoma, is the second most prevalent primary liver cancer after hepatocellular carcinoma (1, 2). ICC is one of the leading causes of cancer-related deaths globally with a dismal prognosis. Currently, surgical resection (SR) is the first-line therapy for ICC. However, only 20% of the patients with ICC are resectable when diagnosed (3). Even the small (≤5 cm) and early-stage ICC might be unresectable due to poor hepatic reserve, complex anatomic location of the lesion, or underlying comorbidities (4). Moreover, almost 80% of patients suffer from a high incidence of complications and loss of liver function due to an extended resection (5). Besides, about 40–70% of patients treated surgically are reported to have positive resection margins with recurrence rates as high as 52% (6). Therefore, effective therapeutic strategies for ICC warrants further attention.

Radiofrequency ablation (RFA) is one of the several ablation therapies for solid tumors. RFA is characterized by its technical ease and minimal invasiveness and has been largely used in the treatment of liver tumors, especially for small early-stage hepatocellular carcinoma (HCC). Several studies and meta-analyses have shown that RFA has similar long-term outcomes and is associated with shorter hospital stays and fewer complications compared to liver resection (7). Therefore, both RFA and hepatectomy have been recommended for the treatment of early-stage HCC (8). Nevertheless, the use of RFA for the treatment of ICC remains controversial. Several studies (case reports and case series) evaluated the effectiveness and safety of RFA in the treatment of advanced or recurrent ICC and affirmed the therapeutic effects of RFA for the small size (≤5 cm) ICC (9–11). However, whether RFA could be applied to a small-size and early-stage primary ICC was unevaluated. Furthermore, information on whether the clinical practice of RFA in HCC could be extrapolated to ICC was unknown. The current guidelines recommend the use of RFA in small and early-stage (T1N0M0) ICC only for patients ineligible for resection (6). This recommendation, however, lacks support from high-level evidence due to the small sample size (*n* < 20) and heterogeneous patient populations (patients with both primary and recurrent ICC). In the present study, we aimed to compare the survival outcomes of RFA and SR for patients with small early-stage primary ICC.

## Methods

### Data Source

Detailed patients' information from 2004 to 2014 was obtained from the Surveillance, Epidemiology, and End Results (SEER) database. SEER is a public database that collects survival and incidence data of various types of cancers and covers more than 25% of the United States' population. SEER data include tumor characteristics such as primary tumor site, TNM staging of the tumor, tumor size, type of treatment, and cause of death, and demographic characteristics such as race, age, sex, etc. We downloaded the data from SEER with SEER^*^Stat Software (version 8.3.4; https://seer.cancer.gov/seerstat/).

### Patients

The present study was designed as a retrospective study. The inclusion criteria were as follows: (1) patients older than 18 years old; (2) patients diagnosed between 2004 and 2014; (3) ICCs identified by topography code C22.0 (primary liver cancer) and histological code 8160 or 8180, or by topography code C22.1 (intrahepatic bile duct cancer) and histological code 8140, 8160, or 8180; (4) patients with small early-stage (T1N0M0) tumors: single lesion, no vascular invasion and extrahepatic extension (collaborative-stage extension codes 100 and 150), without lymph nodes or distant metastasis, and tumor size no more than 5 cm; (5) patients with active follow-up and histological diagnosis; (6) the ICC was the primary or the only tumor diagnosed; and (7) patients underwent RFA (SEER code: 16) or SR (SEER code: 20–25, 30–37, 50–52, 60, 65, and 66). Demographics of patients such as race, age, and marital status, and tumor characteristics such as tumor size, grade, and stage of the tumor were all extracted for subsequent analysis. The primary outcome was overall survival (OS), and secondary outcome was cancer-specific survival (CSS).

### Statistical Analysis

Patients were divided into two groups according to the treatment they received (RFA and SR). The continuous variables were presented as median with interquartile range (IQR). Comparisons between treatment cohorts were analyzed using the Student's *t*-tests or the Mann–Whitney *U*-tests (depending on the normal distribution and multiple testing corrections). Categorical variables were presented as numbers and percentages and compared by chi-square tests or Fisher's exact tests. A *p*-value of < 0.05 was considered statistically significant. Kaplan–Meier survival curves were generated and compared using the log-rank test. A Cox proportional hazards regression analysis was used to compare the survival between RFA and SR. Unadjusted Cox regression analysis was performed followed by the adjusted model. Inverse probability weighting (IPTW) was used to verify the results (12). The adjusted variables include the variables which showed significance in the univariate analysis (*p* < 0.2). Statistical analyses were performed with the Empower software (Waters Corporation, Singapore).

## Results

### Patient and Demographics Details

**Figure 1:**
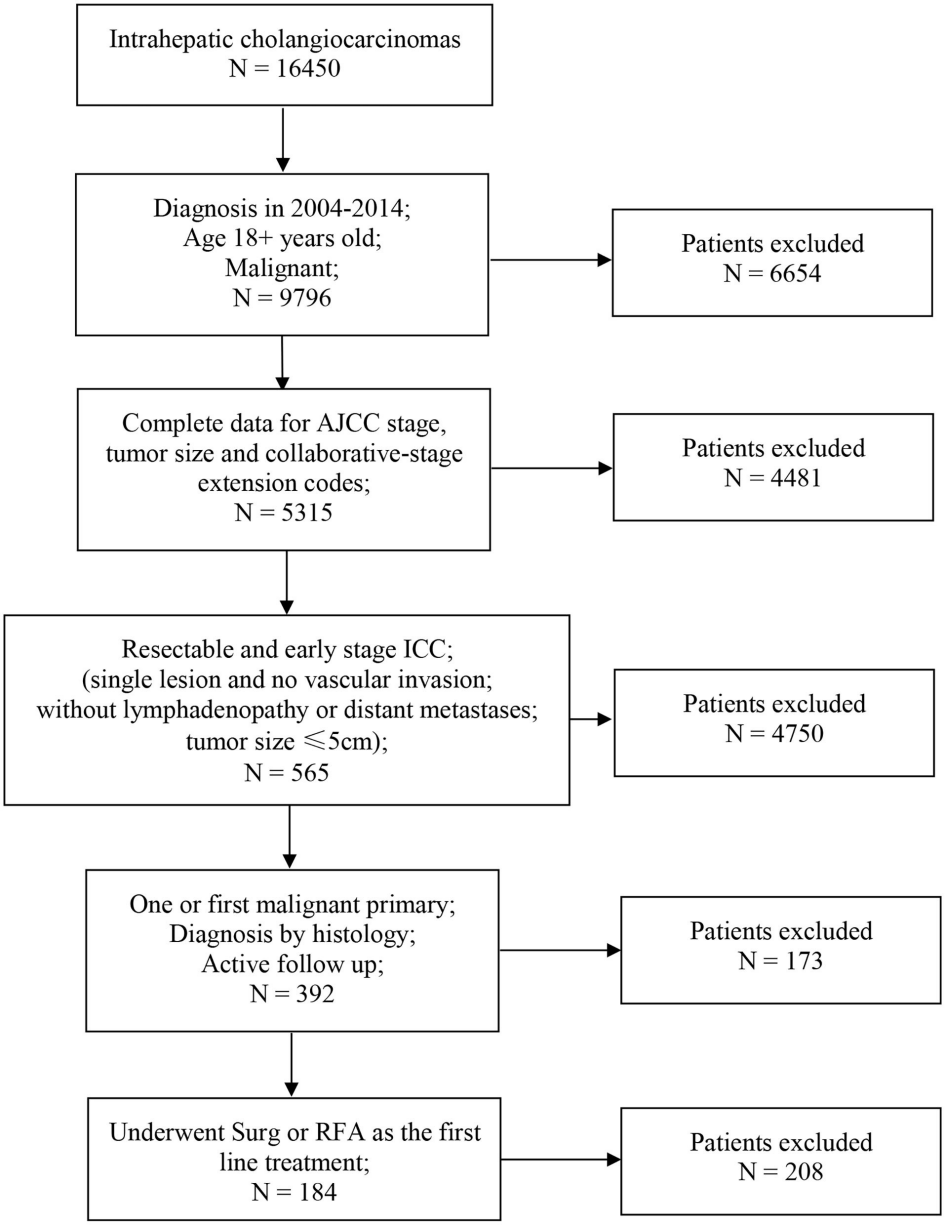
Flowchart of patients selection. AJCC, American Joint Committee on Cancer; ICC, intrahepatic cholangiocarcinoma; Surg, surgical resection; RFA, radiofrequency ablation.

Of the 16,450 patients with a diagnosis of ICC in the SEER database during 2004–2015, 184 patients met the inclusion criteria. The screening procedure is presented in Figure 1. In total, 150 patients received SR treatment, while 34 patients received RFA treatment. Among the SR-treated patients, 68 underwent segmental resection (19 monosegment, 6 two segments, 10 three segments, and 33 unclear segments), 63 underwent hepatic lobectomy (25 right lobectomy, 31 left lobectomy, and 7 unknown side of lobectomy), and 19 underwent the extended lobectomy. The median follow-up for patients of SR group was 32 months (IQR, 26–37), and for RFA group was 31 months (IQR, 25–34). Most of the baseline characteristics for the SR and RFA groups were well balanced (Table 1). The median tumor size in the RFA group was smaller than the SR group. The tumor grade was the only variable with uneven distribution in the two groups (*p* < 0.001). This is because the tumor grade was unknown in the majority of the patients from the RFA group.

**Table 1:** Baseline clinical characteristics for all patients.

**Clinical characteristics**	**Surgical resection**	**Radiofrequency ablation**	***p***
	***n* = 150**	***n* = 34**	
**Race**			0.531
White	27 (18.0%)	9 (26.5%)	
Black	15 (10.0%)	3 (8.8%)	
Other	108 (72.0%)	22 (64.7%)	
**Gender**			0.967
Male	80 (53.3%)	18 (52.9%)	
Female	70 (46.7%)	16 (47.1%)	
**Year of diagnosis**			0.521
2004–2007	38 (25.3%)	7 (20.6%)	
2008–2011	59 (39.3%)	17 (50.0%)	
2012–2014	53 (35.3%)	10 (29.4%)	
**Age at diagnosis**			0.316
≤ 60	53 (35.3%)	10 (29.4%)	
60–70	59 (39.3%)	11 (32.4%)	
>70	38 (25.3%)	13 (38.2%)	
**Marital status at diagnosis**			0.153
Married	94 (62.7%)	17 (50.0%)	
Divorced	18 (12.0%)	5 (14.7%)	
Single	26 (17.3%)	5 (14.7%)	
Widowed	12 (8.0%)	7 (20.6%)	
**Tumor size (cm, median and IQR)**	35.0 (25.0–41.8)	32.0 (24.5–40.0)	0.787
**Tumor grade**			<0.001
Grade I	23 (15.3%)	2 (5.9%)	
Grade II	71 (47.3%)	6 (17.6%)	
Grade III	34 (22.7%)	3 (8.8%)	
Unknown	22 (14.7%)	23 (67.6%)	
**Radiation**			0.299
Yes	12 (8.0%)	1 (2.9%)	
No/Unknown	138 (92.0%)	33 (97.1%)	
**Chemotherapy**			0.671
Yes	36 (24.0%)	7 (20.6%)	
No/Unknown	114 (76.0%)	27 (79.4%)	

### Survival Analysis of Patients With ICC

**Figure 2:**
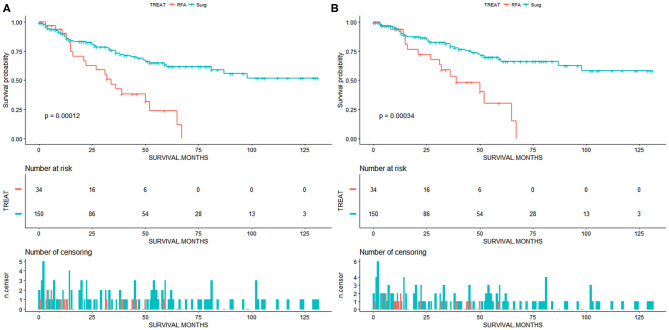
**(A)** Overall survival for patients with small and early-stage intrahepatic cholangiocarcinoma. **(B)** Cancer-specific survival for patients with small and early-stage intrahepatic cholangiocarcinoma. Surg, surgical resection; RFA, radiofrequency ablation.

We perform survival analysis with the overall survival as the primary outcome and the cancer-specific survival as the secondary outcome. The overall survival was measured from the date of resection or RFA to the date of all-cause death. Cancer-specific survival was measured from the date of resection or RFA to the date of death from cancer. In patients with small and early-stage ICC, the SR group had a significantly better OS compared to the RFA group (*p* < 0.001, Figure 2A). The OS rates for the SR group at 1, 3, and 5 years were 87.4, 73.3, and 61.5%, respectively, and 89.9, 42.4, and 23.9%, respectively, for the RFA group (Table 2). After adjusting by the race, marital status, tumor size, and tumor grade, the patients who underwent SR still had a significantly better OS than patients who received RFA (*p* = 0.001). And SR patients had a better CSS than RFA patients (*p* < 0.001, Figure 2B). CSS rates at 1, 3, and 5 years were 91.5, 73.8, and 66.1%, respectively, for the SR group and 93.5, 53.4, and 30.0%, respectively, for the RFA group (Table 3). After adjusting by the year of diagnosis, tumor size, and tumor grade, there was still a significantly better CSS in the SR group than the RFA group (*p* = 0.001). In the inverse probability-weighting model, a significantly better OS and CSS could also be observed in the SR group compared to the RFA group in patients with <5 cm ICC (OS hazard ratio, 2.15; 95% CI: 1.30–3.54; *p* = 0.003 and CSS hazard ratio, 2.22; 95% CI: 1.24–3.98; *p* = 0.07).

**Table 2:** Survival analysis in terms of overall survival.

**Parameters**	**Overall survival**			**Univariate analysis**		**Multivariate analysis**	
				**HR(95%CI)**	***p***	**HR(95%CI)**	***p***
**Primary treatment**	1-year OS	3-year OS	5-year OS		<0.001		0.001
SR	87.4	73.3	61.5	Reference		Reference	
RFA	89.9	42.4	23.9	2.76 (1.61–4.73)		3.26 (1.58–6.51)	
**Race**					0.084		0.698
White	97.1	82.7	70.3	Reference		Reference	
Black	93.3	70.5	52.9	1.27 (0.44–3.68)		1.24 (0.41–3.66)	
Other	84.5	62.4	49.9	1.96 (1.01–3.80)		2.12 (1.08–4.17)	
**Gender**					0.880	
Male	86.4	66.5	56.1	Reference			
Female	89.7	68.7	53.7	0.96 (0.59–1.57)			
**Year of diagnosis**					0.321	
2004–2007	72.2	58.2	46.6	Reference			
2008–2011	88.2	69.5	58.5	0.68 (0.40–1.18)			
2012–2014	85.7	77.2	–	0.58 (0.24–1.42)			
**Age at diagnosis**					0.325	
≤ 60	89.6	64.9	56.6	Reference			
60–70	86.2	72.2	59.1	0.79 (0.43–1.44)			
>70	81.0	65.3	46.7	1.28 (0.71–2.28)			
**Marital status**					0.082		0.803
Married	89.7	73.2	56.9	Reference		Reference	
Divorced	90.0	73.4	64.2	0.79 (0.33–1.88)		0.89 (0.37–2.14)	
Single or widowed	83.1	52.3	45.8	1.11 (0.54–2.24)		1.07 (0.52–2.21)	
**Tumor size (cm)**					0.032		0.046
<3	85.6	75.4	62.9	Reference		Reference	
3–5	89.6	61.2	48.4	1.60 (1.26–2.39)		1.71 (1.01–2.90)	
**Tumor grade**					0.051		0.295
Grade I–II	93.4	72.7	65.2	Reference		Reference	
Grade III	85.5	61.1	55.5	1.42 (0.73–2.76)		1.42 (0.73–2.77)	
Unknown	85.7	61.2	44.7	1.99 (1.15–3.45)		1.25 (0.66–2.37)	
**Radiation**					0.271		
Yes	83.3	75.0	66.7	Reference			
No/unknown	88.3	66.6	53.2	1.69 (0.61–4.65)			
**Chemotherapy**					0.932		
Yes	80.3	63.8	59.5	Reference			
No/unknown	90.0	67.3	53.3	1.02 (0.56–1.85)			

*HR, hazard ratio; CI, confidence interval; SR, surgical resection; RFA, radiofrequency ablation; OS, overall survival*.

**Table 3:** Survival analysis in terms of cancer-specific survival.

**Parameters**	**Cancer-specific survival**			**Univariate analysis**		**Multivariate analysis**	
				**HR(95%CI)**	***p***	**HR(95%CI)**	***p***
**Primary treatment**	1-year CSS	3-year CSS	5-year CSS		<0.001		0.001
SR	91.5	73.8	66.1	Reference		Reference	
RFA	93.5	53.4	30.0	2.87 (1.57–5.24)		3.82 (1.72–8.44)	
**Race**					0.557		
White	–	89.0	75.6	Reference			
Black	93.3	75.5	56.6	1.43 (0.43–4.76)			
Other	88.4	69.7	52.6	2.27 (1.06–4.89)			
**Gender**					0.695		
Male	92.8	75.8	64.0	Reference			
Female	90.7	72.6	54.7	1.11 (0.64–1.92)			
**Year of diagnosis**					0.058		0.341
2004–2007	82.6	71.4	57.1	Reference		Reference	
2008–2011	94.7	74.7	61.6	0.92 (0.49–1.72)		0.73 (0.38–1.38)	
2012–2014	89.5	80.5	–	0.70 (0.24–2.03)		0.65 (0.22–1.88)	
**Age at diagnosis**					0.161		
≤ 60	94.9	68.1	59.4	Reference			
60–70	93.9	81.3	66.5	0.60 (0.30–1.22)			
>70	85.0	70.5	53.1	1.19 (0.63–2.24)			
**Marital status**					0.713		
Married	92.6	78.9	61.3	Reference			
Divorced	–	77.3	67.6	0.83 (0.32–2.15)			
Single or widowed	86.2	62.3	54.6	1.52 (0.83–2.76)			
**Tumor size (cm)**					0.028		0.041
<3	90.0	82.0	68.4	Reference		Reference	
3–5	92.5	73.2	53.8	1.68 (1.24–2.72)		1.86 (1.02–3.38)	
**Tumor grade**					0.035		
Grade I–II	93.4	76.6	68.8	Reference		Reference	
Grade III	91.2	69.8	63.4	1.36 (0.84–2.66)		1.33 (0.63–2.81)	
Unknown	90.6	72.4	43.7	1.93 (1.05–3.56)		1.17 (0.58–2.36)	
**Radiation**					0.568		
Yes	83.3	75.0	66.7	Reference			
No/unknown	92.6	74.0	59.1	1.34 (0.48–3.73)			
**Chemotherapy**					0.573		
Yes	82.9	69.6	61.5	Reference			
No/unknown	94.5	75.6	59.8	0.83 (0.44–1.56)			

*HR, hazard ratio; CI, confidence interval; SR, surgical resection; RFA, radiofrequency ablation; CSS, cancer-specific survival*.

The effectiveness of RFA was affected by the tumor size, and for the evaluation of whether tumor size smaller than 5 cm would have influence on the above results, we performed a stratification analysis based on the tumor size. As showed in Supplementary Tables 1, 2, the SR group had a significantly better OS and CSS than the RFA group in patients of tumor size <4.5 cm (*p* < 0.001, Supplementary Figures 1A,D). Additionally, in patients with tumor size <4.5 or 4.0 cm, the OS and CSS of patients from SR group were still significantly better than that of patients from the RFA group (*p* < 0.05 in Supplementary Figures 1B,C,E,F).

**Figure 3:**
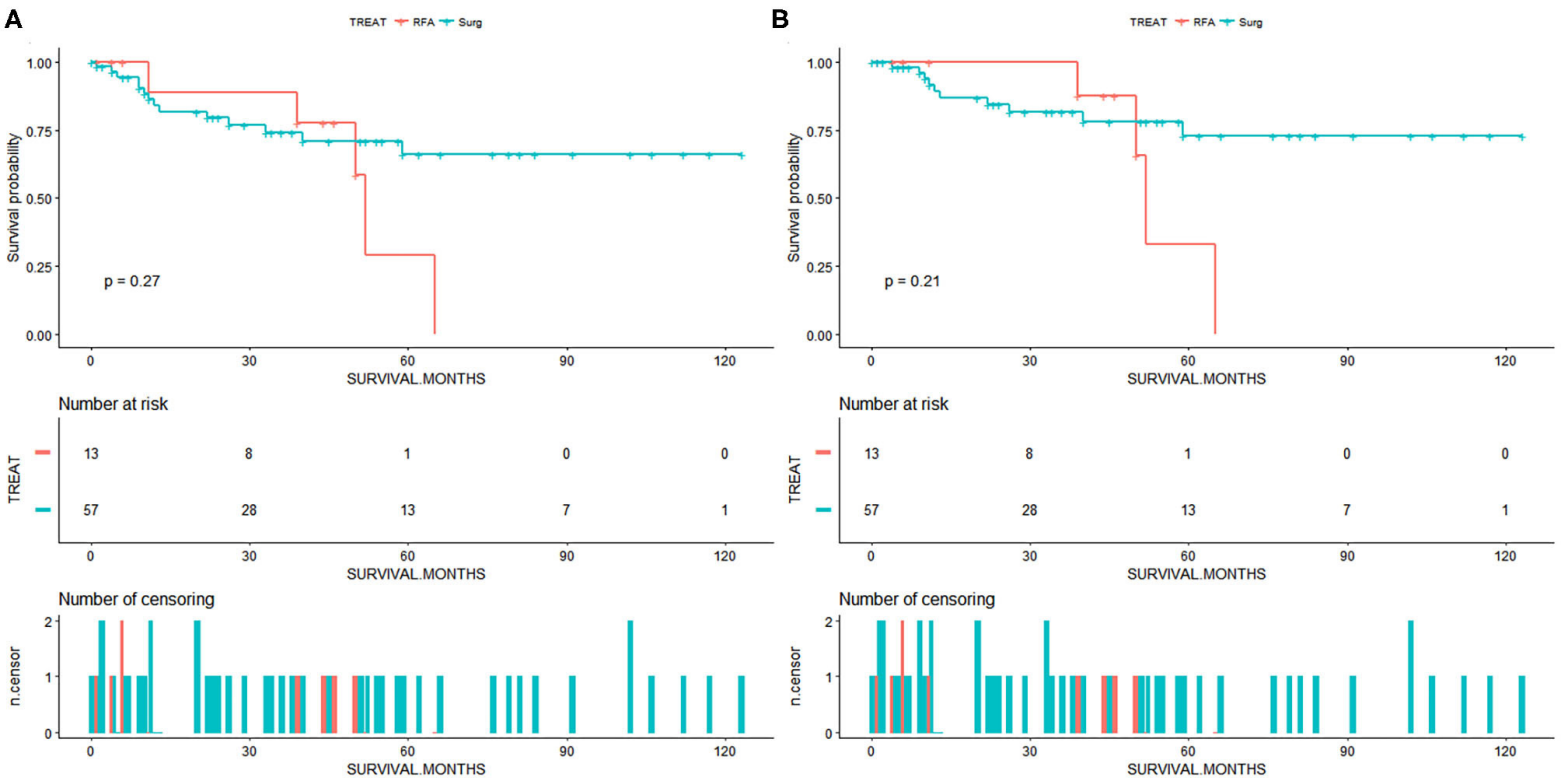
**(A)** Overall survival for patients with intrahepatic cholangiocarcinoma of tumor size <3 cm. **(B)** Cancer-specific survival for patients with intrahepatic cholangiocarcinoma of tumor size <3 cm. Surg, surgical resection; RFA, radiofrequency ablation.

Due to a low sample size (<20 patients in the RFA group), the multivariate Cox and IPTW analysis was not performed in patients with <3.5 or 3 cm ICC. For patients with <3 cm ICC, we draw the survival curves and calculated the survival rate of patients from the SR and RFA groups (Figure 3). The OS rates at 1, 3, and 5 years were 84.0, 74.2, and 66.2%, respectively, for the SR group and 88.9, 77.8, and 58.3%, respectively, for the RFA group. The median survival was 38 months for the SR group and 39 months for RFA group. The CSS rates at 1, 3, and 5 years were 89.2, 81.7, and 72.9%, respectively, for the SR group and 89.5, 87.5, and 62.5%, respectively, for the RFA group. Among the RFA patients, the 5-year OS rate improved from 23.9 to 58.3% after patients with tumor size >3 cm were excluded from analysis (Supplementary Table 1). The 5-year CSS rate also improved from 30.0 to 62.5% (Supplementary Table 2). There might be a comparable survival rate between the SR and RFA groups; however, the non-inferiority analysis was not allowed due to the low sample size in this comparison.

## Discussion

At present, although the technological advancements in the diagnosis and surgical resection of malignant tumors are underway, the incidence rates and mortality rates of ICC are intensifying worldwide (6). Therefore, innovative alternative techniques for the treatment of ICC are essential. RFA is an alternative liver-targeted therapy, which applies heat to destroy a tumor without damaging the surrounding liver tissue. Recent studies on HCC have shown that RFA can achieve results similar to hepatectomy in the treatment of small early-stage HCC. However, the feasibility of RFA in the treatment of ICC is still controversial.

In 2002, Slakey (13) reported the first case of ICC treatment with RFA. The results showed that RFA might increase the surgical resection rate of patients. Recently, several studies have demonstrated RFA treatment for patients with recurrent ICC. For instance, Kamphues et al. (14) assessed 13 ICC patients who underwent repeated resection and RFA. They found that repeated resection combined with RFA was safe and effective, and the median survival time of these patients was 51 months. Fu et al. evaluated the effectiveness of RFA for the treatment of patients with recurrent ICC. The median overall survival was 30 months for the 12 patients who lost the chance for the repeated resection (11). In another study by Kim et al. (15), the technical effectiveness rate of RFA in the treatment of recurrent ICC reached 97%, and the median survival rate after RFA was 27.4 months. Repeated resection was suggested to be the acceptable technique in the treatment of recurrent ICC. Other studies by Ohtsuka et al. (16) and Song et al. (17) reported that repeated surgical resection of recurrent ICC can provide long-term survival opportunities for patients. However, for patients with poor liver reserve function, the effectiveness of repeated surgical resection was not favorable. Zhang et al. compared the efficacy of RFA and repeated surgical resection in patients with recurrent ICC. In this study, 109 patients with recurrent ICC underwent repeated surgical resection or thermal ablation after radical surgery (18). The results showed that the OS rate of repeated resection patients was similar to that of thermal ablation patients. Thermal ablation can be an effective treatment for recurrent ICC, but its use should be limited to tumors <3 cm in diameter (18). This study was one of the few studies comparing SR and RFA for the treatment of ICC.

Several studies have assessed the efficacy of RFA in the treatment of advanced ICC. Chiou et al. (19) evaluated the perioperative outcomes of RFA in the treatment of advanced ICC with 10 patients. Carrafiello et al. (9) shared their preliminary experiment of the RFA for the unresectable ICC, and the short-term follow-up results confirmed the applicability of RFA. Kim et al. (20) found that RFA offered a successful tumor control in the treatment of 13 patients with 17 advanced ICC; the median survival rate reached 38.5 months. While the median survival rate of resection ICC patients was 27.6 months, it was only 12.9 months for the non-surgical candidates with varied palliative treatment in a study by Dhanasekaran et al. (21). Recently, Wu et al. (22) found that for patients with unresectable ICC, RFA was associated with significantly better survival rates compared with chemoradiotherapy. RFA was recommended as one of the standard treatments for ICC in the European Association for the Study of the Liver (EASL) guidelines, although the evidence level was only C2 (6).

Previously, RFA has been recommended for patients with early-stage ICC where surgery is not an option. However, this recommendation lacked verification due to the lack of studies on the use of RFA for the treatment of primary early-stage ICC. In the present study, 184 patients with small T1 stage ICC who received either RFA or SR were selected. The prognosis of patients who received different treatment modalities was compared. Our results showed that SR provided a significantly better survival rate than RFA in patients with small and early-stage ICC (T1N0M0, tumor size <5 cm). And whether the RFA could provide a comparable prognosis benefit as the SR in patients with <3 cm ICC should be explored in future studies, although a similar median survival was observed in the present study. The 5-year OS rate after the RFA treatment for patients with <3 cm ICC reached 58.3%. The size of the tumor had a great effect on the implementation of RFA. Generally, the complete necrosis of a tumor bigger than 5 cm is difficult to achieve in a single RFA session, as there is always a residual tumor. Therefore, only patients with a tumor <5 cm are screened for RFA treatment studies. In studies by Carrafiello et al. (9) and Kim et al. (15), patients with <3 cm ICC had less residual tumor, lower recurrent rate, and almost no complications after the RFA treatment. The difference between the geometry of the tumor and the necrosis induced by RFA always leads to insufficient ablation of larger tumors. Although multimodality RFA has been considered a feasible method for the treatment of large tumors, its application in the treatment of ICC is limited (23). Other factors that affect the success rate of RFA are tumor location and surrounding tissue (24). The central tumor is more difficult to be successfully treated because of the heat loss caused by the extensive blood vessels in the liver hilum. In the process of RFA, heat loss occurs at the tip of the needle, mainly through blood circulation. Difficulties in the ablation of such tumors may be overcome by stereotactic planning of multiple overlapping ablations, which should be explored in future studies.

In addition to the inherent limitations of the retrospective study such as the selective basis, there were other limitations in the study. First, although a population-based database was utilized to screen patients, the sample size in our study was still small; all the comparisons that included low sample size for survival analysis should be verified in future studies. Second, information for adjuvant chemotherapy and radiotherapy in the survival analysis did not contain the details of the protocols, which was also not available in the SEER database. Third, disease-free survival could not be determined due to the lack of information on the local recurrence in the SEER database. Fourth, the liver function and other comorbidities of the patients were not assessed. Fifth, different types of resections and tumor locations might have led to bias. Sixth, stereotactic RFA patients could not be identified due to the limitation of the SEER, although stereotactic RFA was suggested to be feasible for large-size tumors or subcapsular tumor location. Finally, we could not obtain data on surgical margin status in the SEER; surgical margin status was an important prognostic factor in patients with resectable ICC.

## Conclusion

The present study illustrated that the SR provided a significantly better prognosis than RFA in patients with small and early-stage ICC (T1N0M0, tumor size <5 cm). SR as the first-line treatment of primary early-stage ICC is still recommended. Whether the RFA could provide a comparable prognosis benefit as the SR in patients with <3 cm ICC should be explored in future studies with more included patients. Prospective randomized controlled trials with larger sample sizes are necessary to compare these modalities in the treatment of ICC.

## Data Availability Statement

Publicly available datasets the Surveillance, Epidemiology, and End Results (SEER) database were analyzed in this study. This data can be found here: https://seer.cancer.gov/seerstat/.

## Author Contributions

XX, ZJ, DH, and HL collected, extracted, and analyzed the data and wrote the paper. PL and HL conceived and designed this study. All authors reviewed the paper, read, and approved the final manuscript.

## Conflict of Interest

The authors declare that the research was conducted in the absence of any commercial or financial relationships that could be construed as a potential conflict of interest.
