# Supplementary material for: Radiofrequency Ablation vs. Surgical Resection for Small Early-Stage Primary Intrahepatic Cholangiocarcinoma
Source: Front Oncol. 2020 Sep 29;10:540662. doi: 10.3389/fonc.2020.540662 (PMC7550467; doi:10.3389/fonc.2020.540662)
Supplement: Supplementary file 1 [file Data_Sheet_1.docx]

**Supplementary Figure 1.** Overall survival for patients with intrahepatic cholangiocarcinoma of tumor size A)＜4.5cm; B)＜4cm; C)＜3.5cm. Cancer-specific survival for patients with intrahepatic cholangiocarcinoma of tumor size D)＜4.5cm; E)＜4cm; F)＜3.5cm. Surg, Surgical resection; RFA, radiofrequency ablation.


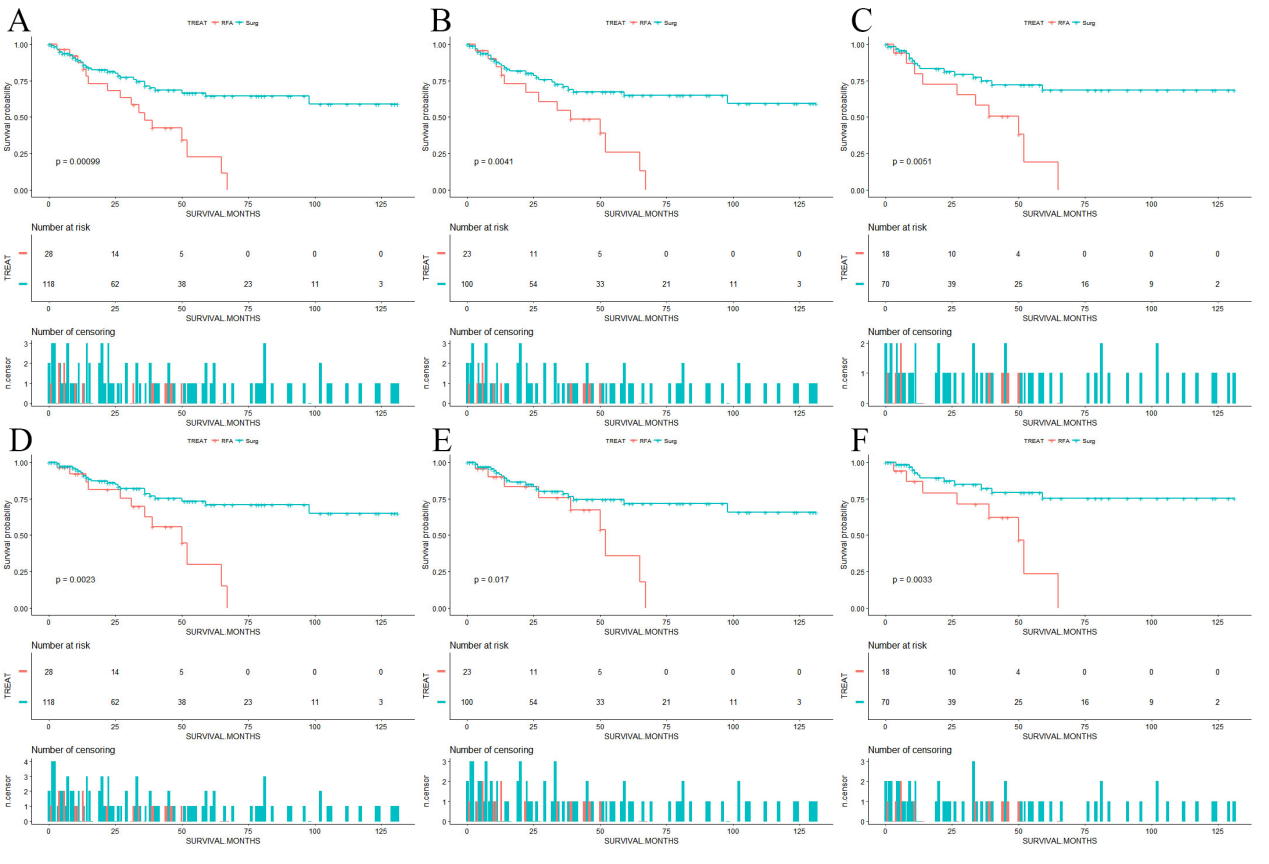


**Supplementary Table 1. Comparison between the resection and radiofrequency ablation in terms of overall survival**

| **Tumor**  **Size** | **Sample**  **Size** | **OS** | | | **HR（95%CI）** | **Unadjusted**  **p** | **HR^a^（95%CI）** | **Adjusted**  **p^a^** | **HR^b^（95%CI）** | **Adjusted**  **p^b^** |
| --- | --- | --- | --- | --- | --- | --- | --- | --- | --- | --- |
| **＜4.5cm** | 146 | 1-yr OS | 3-yr OS | 5-yr OS |  | 0.001 |  | 0.016 |  | 0.002 |
| **Surg** | 118 | 87.7 | 71.4 | 64.4 | Ref |  | Ref |  | Ref |  |
| **RF** | 28 | 87.3 | 47.8 | 22.7 | 2.67 (1.45-4.93) |  | 2.76 (1.21-6.30) |  | 2.30 (1.35-3.94) |  |
| **＜4cm** | 123 |  | | |  | 0.005 |  | 0.047 |  | 0.007 |
| **Surg** | 100 | 86.4 | 70.8 | 64.9 | Ref |  | Ref |  | Ref |  |
| **RF** | 23 | 84.5 | 54.6 | 25.9 | 2.57 (1.31-5.01) |  | 2.61 (1.01-6.71) |  | 2.13 (1.22-3.72) |  |
| **＜3.5cm** | 88 |  | | |  | - |  | - |  | - |
| **Surg** | 70 | 85.1 | 74.7 | 68.5 | - |  | - |  | - |  |
| **RF** | 18 | 79.6 | 57.9 | 19 | - |  | - |  | - |  |
| **＜3cm*** | 70 |  | | |  | - |  | - |  | - |
| **Surg** | 57 | 84.0 | 74.2 | 66.2 | - |  | - |  | - |  |
| **RF** | 13 | 88.9 | 77.8 | 58.3 | - |  | - |  | - |  |

^a^, the multivariable cox model; ^b^, inverse probability weighting model; OS, overall survival; HR, hazard ratio; CI, confidence interval; Surg, surgical resection; RF, radiofrequency ablation. *, the sample size was low, so that the cox analysis was not performed.

**Supplementary Table 2. Comparison between the resection and radiofrequency ablation in terms of cancer-specific survival**

| **Tumor**  **Size** | **Sample size** | **CSS** | | | **HR（95%CI）** | **Unadjusted**  **p** | **HR^a^（95%CI）** | **Adjusted**  **p^a^** | **HR^b^（95%CI）** | **Adjusted**  **p^b^** |
| --- | --- | --- | --- | --- | --- | --- | --- | --- | --- | --- |
| **＜4.5cm** | 146 | 1-yr CSS | 3-yr CSS | 5-yr CSS |  | 0.003 |  | 0.015 |  | 0.004 |
| **Surg** | 118 | 93.0 | 78.6 | 70.8 | Ref |  | Ref |  | Ref |  |
| **RF** | 28 | 91.9 | 62.6 | 29.7 | 2.87 (1.41-5.85) |  | 3.62 (1.28-10.23) |  | 2.57 (1.35-4.90) |  |
| **＜4cm** | 123 |  | | |  | 0.021 |  | 0.044 |  | 0.022 |
| **Surg** | 100 | 91.7 | 78.3 | 71.8 | Ref |  | Ref |  | Ref |  |
| **RF** | 23 | 90.2 | 75.4 | 35.9 | 2.56 (1.15-5.69) |  | 3.44 (1.03-11.44) |  | 2.20 (1.11-4.36) |  |
| **＜3.5cm*** | 88 |  | | |  | - |  | - |  | - |
| **Surg** | 70 | 91.1 | 82.2 | 75.4 | - |  | - |  | - |  |
| **RF** | 18 | 86.9 | 71.1 | 23.3 | - |  | - |  | - |  |
| **＜3cm*** | 70 |  | | |  | - |  | - |  | - |
| **Surg** | 57 | 89.2 | 81.7 | 72.9 | - |  | - |  | - |  |
| **RF** | 13 | 89.5 | 87.5 | 62.5 | - |  | - |  | - |  |

^a^, the multivariable cox model; ^b^, inverse probability weighting model; CSS, cancer-specific survival; HR, hazard ratio; CI, confidence interval; Surg, surgical resection; RF, radiofrequency ablation. *, the sample size was low, so that the cox analysis was not performed.
